# Exogenous Application of a Plant Elicitor Induces Volatile Emission in Wheat and Enhances the Attraction of an Aphid Parasitoid *Aphidius gifuensis*

**DOI:** 10.3390/plants11243496

**Published:** 2022-12-13

**Authors:** Dianzhao Xiao, Jiahui Liu, Yulong Liu, Yiwei Wang, Yidi Zhan, Yong Liu

**Affiliations:** 1 College of Plant Protection, Shandong Agricultural University, No. 61 Daizong Road, Taian 271018, China; 2 Department of Functional and Evolutionary Entomology, University of Liège, Gembloux Agro-Bio Tech, Passage des Déportés 2, 5030 Gembloux, Belgium

**Keywords:** wheat aphid, HIPVs, indirect defense, methyl salicylate, biological control

## Abstract

It is well known that plant elicitors can induce plant defense against pests. The herbivore-induced plant volatile (HIPV) methyl salicylate (MeSA), as a signaling hormone involved in plant pathogen defense, is used to recruit natural enemies to protect wheat and other crops. However, the defense mechanism remains largely unknown. Here, the headspace volatiles of wheat plants were collected and analyzed by gas chromatography-mass spectrometry (GC−MS), gas chromatography with electroantennographic detection (GC−EAD) and principal component analysis (PCA). The results showed that exogenous application of MeSA induced qualitative and quantitative changes in the volatiles emitted from wheat plants, and these changes were mainly related to Carveol, Linalool, m-Diethyl-benzene, p-Cymene, Nonanal, D-limonene and 6-methyl-5-Hepten-2-one. Then, the electroantennogram (EAG) and Y-tube bioassay were performed to test the physiological and behavioral responses of *Aphidius gifuensis* Ashmesd to the active volatile compounds (p-Cymene, m-Diethyl-benzene, Carveol) that identified by using GC-EAD. The female *A. gifuensis* showed strong physiological responses to 1 μg/μL p-Cymene and 1 μg/μL m-Diethyl-benzene. Moreover, a mixture blend was more attractive to female *A. gifuensis* than a single compound. These findings suggested that MeSA could induce wheat plant indirect defense against wheat aphids through attracting parasitoid in the wheat agro-ecosystem.

## 1. Introduction

In the arms race against herbivory, plants have evolved a variety of defense mechanisms including constitutive defense and inductive defense. Induced plant defense, which only respond when plants feel herbivore attack, is an important mechanism for plants to resist herbivores [1,2]. The volatile organic compounds (VOCs) emitted after herbivore attack play a significant role in plant defense, and the potential of manipulating their emission has been raised to enhance crop protection [3,4,5]. Upon herbivory attack, plants emit herbivore-induced plant volatiles (HIPVs) or oviposition-induced plant volatiles (OIPVs), which could be perceived by natural enemies to locate their host [6]. The production of HIPVs depends on metabolic pathways activated by different compounds such as phytohormones or their derivatives. These compounds can act as elicitors of HIPVs that have no direct toxicity to insects, but can induce plant defensive responses [7].

Salicylic acid (SA) and methyl salicylate (MeSA) are common in some plants and SA is easily converted to MeSA by enzymatic methylation reaction [8]. MeSA is a volatile compound associated with the salicylic acid pathway in plants and has been shown to act as a mobile signal for systemic acquired resistance (SAR) by being converted to SA [9]; it is known to promote the expression of defense related genes in response to herbivores and pathogens [10]. Spraying MeSA on the leaves of citrus seedlings can induce the underground part to produce a specific volatile D-limonene, which can attract the natural enemy nematode *Steinernema diaprepesi* [11]. Poplar tree leaves treated with MeSA exhibit induced expression of defense genes and increased emission of volatile compounds [12]. Additionally, MeSA-treated Lima bean plants also exhibit elevated emission of two homo-terpenes, which are able to attract foraging predatory mites [13,14]. Application of slow-release MeSA alginate beads in wheat fields can reduce the abundance of *Sitobion avenae* by attracting predators and parasitoids [15,16]. These results suggest that MeSA might attract natural enemies directly and/or indirectly through inducing the emission of plant volatiles.

Wheat is one of the main grain products responsible for supporting the world’s human population [17]. *Sitobion miscanthi* (Takahashi) is a major insect pest of wheat, which reduces crop yield and quality annually [18]. Because of the urgent need for sustainable agriculture and for reducing reliance on pesticide, increasing attention has been paid to conservation practices that seek to increase the biological control efficacy in agroecosystems [19,20]. *Aphidius gifuensis* is widely used in biological control and plays an important role in inhibiting aphid populations [16,21]. 

Our previous studies demonstrated that MeSA applied in wheat fields could enhance the biocontrol efficacy of aphids by attracting their natural enemies [15,16], but the defense mechanism is still poorly understood. We hypothesized that MeSA exposure could alter the volatile emission in wheat and thus perform indirect defense by attracting the parasitic wasp *A. gifuensis*. Here, we collected and analyzed wheat plant volatiles treated with MeSA with gas chromatography-mass spectrometry (GC−MS), gas chromatography with electroantennographic detection (GC−EAD) and principal component analysis (PCA), Electrophysiological and behavioral assays were conducted to test the responses of *A. gifuensis* to the putative volatiles so as to obtain insight into the mechanism of MeSA application for wheat defense against aphids. 

## 2. Results

### 2.1. Olfactory Responses of A. gifuensis to Wheat Plants and Plant Extracts 

**Figure 1:**
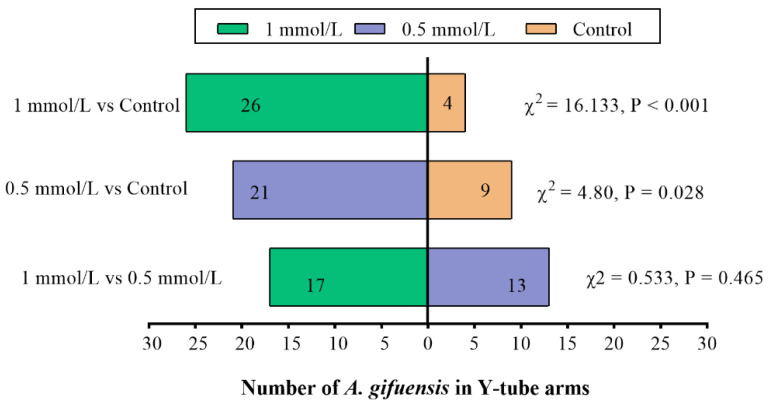
Responses of female *Aphidius gifuensis* (n = 30) in a Y-tube olfactometer between the arm with the untreated plants (control) and the arm treated with either 1 mmol/L MeSA or the 0.5 mmol/L MeSA plants.

**Figure 2:**
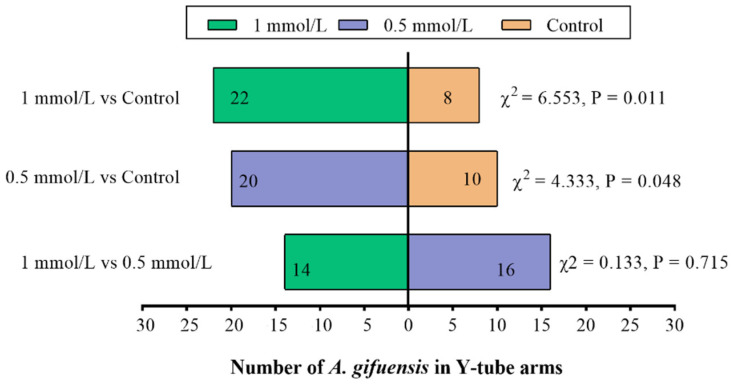
Responses of female *Aphidius gifuensis* (n = 30) in a Y-tube olfactometer between the arm with the untreated plant extracts and the arm treated with either 1 mmol/L MeSA or the 0.5 mmol/L MeSA plant extracts.

In order to demonstrate the responses of *A. gifuensis* to wheat plants and the plant extracts treated with MeSA (0.5 mmol/L MeSA, 1 mmol/L MeSA and control), the Y-tube olfactometer tests were performed. Compared with the control, both 1 mmol/L MeSA and 0.5 mmol/L MeSA treated plants (Figure 1) as well as the extracts from treated plants (Figure 2) showed significant attraction to female *A. gifuensis.* The results showed that the wheat plants or plant extracts could release VOCs that attracted female *A. gifuensis* after MeSA treatment.

### 2.2. Analysis of Wheat Seedling Volatiles

A total of 20 VOCs were detected in the volatile profiles of both untreated and treated plants (Table 1). The identified compounds were primarily alcohols, aldehydes, ketones, terpenes and alkanes. Qualitative differences were observed in volatile emission between untreated and treated plants. Among the 20 volatile compounds identified, 13 volatile compounds were identified induced by MeSA and seven volatile compounds were unique to 1 mmol/L: 2,5-Dimethyl-1-hepten-4-ol, p-Cymene, Carveol, Levomenthol, Linalool, Tetradecane and β-Acorenol. (Table 1).

Eleven volatile compounds were selected from the identified volatiles and the relative content changes of different treatments were analyzed at 24, 48 and 72 h (Table 2). The relative contents of 3-carene and 6-methyl-5-Hepten-2-one in 1 mmol/L MeSA treatment were significantly higher than that in 0.5 mmol/L MeSA treatment. Linalool, Carveol, m-Diethyl-benzene and Levomenthol were emitted only from the 1 mmol/L treatment. The emission of D-Limonene significantly increased at 24, 48 and 72 h compared with the control and the emission of MeSA at 72 h was significantly higher than that at 24 and 48 h.

### 2.3. Multivariate Analysis VOCs 

**Figure 3:**
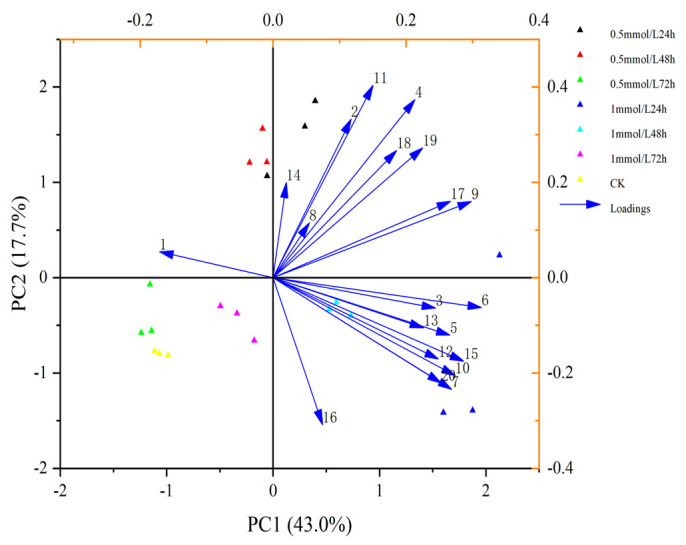
Principal Component Analysis (PCA) of the 20 volatile organic compounds (VOCs) emit-ted from wheat plant. The numbers represented the chemical compounds were as showed in Table 1. Scatter plots visualize the location of each collected sample on each PC with the percentage of explained variation in parentheses, whereas vectors (blue line) visualize the loadings for each VOC.

**Figure 4:**
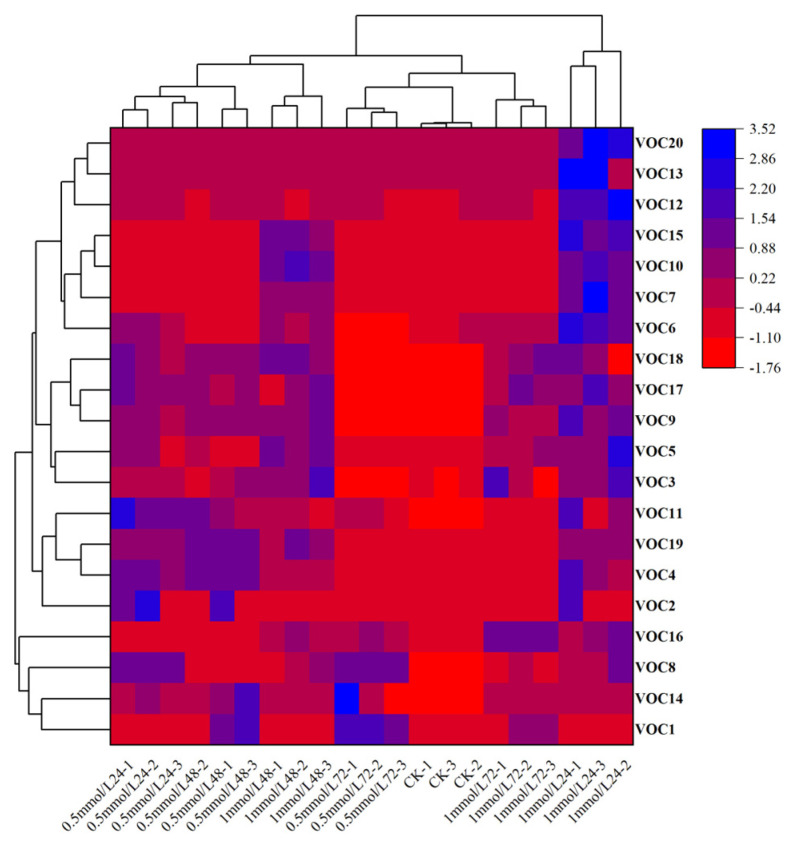
Heatmap clustering showing the abundance (in decreasing color intensity) of the most discriminant VOCs across replicates of wheat plants. Alcohols (20, 13, 10, 15, 2), aldehyde (12, 14, 17, 11), hydrocarbon (8, 14, 1, 7, 6, 18, 9, 3, 19, 4), keto (5), ester (16). The numbers represented the chemical compounds were as showed in Table 1.

A multivariate statistical technique, principal component analysis (PCA). was used for elucidating how each of these 20 VOCs contributed to the variation in the blends obtained from different wheat treatments. The first and second principal components explained 43.0% and 17.7% of the total variance, respectively. PCA showed that these changes were mainly related to Carveol, Linalool, m-Diethyl-benzene, p-Cymene, Nonanal, D-limonene and 6-methyl-5-Hepten-2-one (Figure 3). Heatmap clustering was performed to illustrate variations in VOCs across replicates of untreated and treated plants with MeSA and the findings showed that most of the discriminant VOCs were abundant in wheat plants treated with 1 mmol/L MeSA (Figure 4).

### 2.4. Gas Chromatography-Electroantennogram Detection 

**Figure 5:**
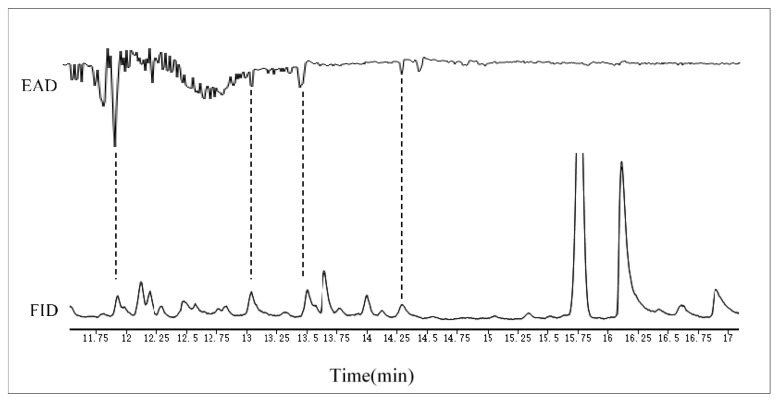
Coupled GC-EAD analysis showing antennal responses of female *Aphidius gifuensis* to volatile organic compound samples collected from wheat plants that were treated with MeSA (1 mmol/L). Upper trace: antennal response, lower trace: FID response. The EAD-active VOCs for female *A. gifuensis* were identified as: m-Diethyl-benzene, p-Cymene, Carveol and Linalool.

GC-EAD analysis showed that female *A. gifuensis* had electrophysiological responses to 4 compounds in MeSA treated samples (Figure 5). Electrophysiological responses related to peaks were demonstrated, with compounds tentatively identified by GC-MS as m-Diethylbenzenel, p-Cymene, Carveol and Linalool.

### 2.5. EAG Responses to Selected Compounds

**Figure 6:**
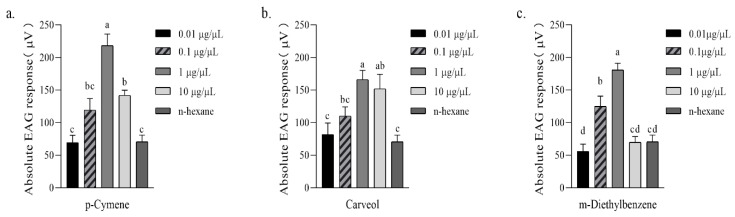
Absolute EAG response [mean ± SE (μV), n = 6] of female *Aphidius gifuensis* antenna to 10 μL of synthetic chemical. (**a**) P-cymene, (**b**) Carveol, (**c**) M-Diethylbenzenel. Different letters indicate that values differ statistically at *p* < 0.05 (ANOVA, followed by LSD test).

Compared with n-hexane (control), female *A. gifuensis* showed significant EAG responses to 1 μg/μL p-Cymene (218.50 ± 17.76 μV), 10 μg/μL p-Cymene (142.17 ± 7.72 μV), 1 μg/μL Carveol (166.30 ± 14.11 μV), 10 μg/μL Carveol (152.00 ± 22.31 μV), 0.1 μg/μL m-Diethyl-benzene (125.17 ± 15.57 μV) and 1 μg/μL m-Diethyl-benzene (180.67 ± 10.55 μV) (Figure 6).

### 2.6. Y-Tube Olfactometer Bioassay 

**Figure 7:**
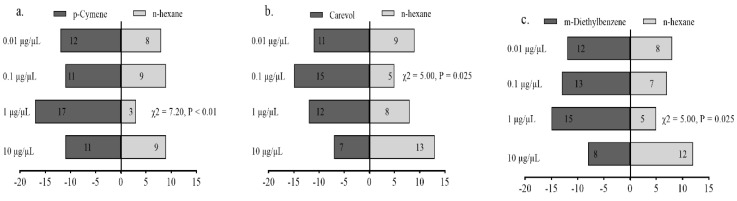
Behavioral responses of *Aphidius gifuensis* female adults (n = 20) to 10 μL of synthetic chem-icals at different concentrations (0.01–10 μg/μL) in olfactometer bioassays: (**a**) p-Cymene, (**b**) Carveol, (**c**) m-Diethylbenzenel.

**Figure 8:**
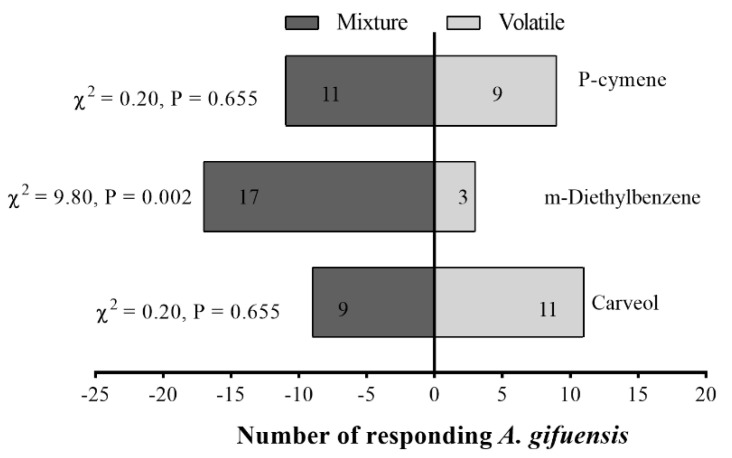
Responses of female *Aphidius gifuensis* (n = 20) in a Y-tube olfactometer to the single com-pound against mixture. The mixture was prepared from 1 μg/μL p-Cymene, 0.1 μg/μL Carveol and 1 μg/μL m-Diethyl-benzene, the concentration of the single compound in the mixture was kept con-stant. Recipe for 1000 μL of mixture: 1.16 μL p-Cymene, 1.16 μL m-Diethyl-benzene, 0.104 μL Carveol, 997.58 μL n-hexane.

Female adults of *A. gifuensis* were significantly attracted to 1 μg/μL p-Cymene (Figure 7a), 0.1 μg/μL Carveol (Figure 7b), and 1 μg/μL m-Diethyl-benzene compared with the control (Figure 7c). The mixed blend was significantly attractive to female *A. gifuensis* compared with m-Diethyl-benzene (Figure 8).

## 3. Discussion

The grain aphid, *S. miscanthi*, is one of the most destructive pests of wheat in China [20,22]. *A. gifuensis* is the dominant parasitoid species in wheat fields and plays an important role in inhibiting the population growth of wheat aphids [16,21].

In plants, S-adenosyl-L-methionine (SAM)-dependent methyltransferases (MTs) can methylate the carboxyl group of SA [8]. Similar to glycosylation, methylation inactivates SA for its function and, reversibly, MeSA can be converted into active SA by the methyl-esterases (MESs) [9,23]. In previous studies, treating plants with exogenous SA can increase the activity of insect resistance enzymes in plants [24]. This produces a large number of secondary metabolites that inhibit the growth and development of pests [25] and induces the defense ability of plants against pathogens [26]. Similar to the report on poplar plants after being sprayed with MeSA [27], in this study wheat plants treated with MeSA showed significant attraction to *A. gifuensis*. After treating with MeSA, the composition of volatiles in wheat leaves was changed and the new terpene volatiles were produced. Studies have shown that a large part of terpenes can be used to regulate the interactions among plants, pests and natural enemies [28]. We found that different concentrations of MeSA induced different volatile profiles in wheat leaves. At lower concentrations (0.5 mmol/L MeSA), only a few volatiles were emitted after induction. Some volatiles such as Carveol, Linalool and Levomenthol were only emitted when exposed to 1 mmol/L MeSA. There may be a minimum dose threshold of exogenous elicitors in inducing plant resistance [29,30]. 

GC-MS results showed that the volatile compounds and the release amount reached the maximum at 24 h after treated with 1 mmol/L and 0.5 mmol/L MeSA, and decreased gradually at 48 h and 72 h. However, D-limonene and MeSA emissions dramatically increased at 72 h. This may have opened up the de novo synthesis pathway of MeSA. This pathway could be opened after wheat was treated with MeSA and the release amount of MeSA was gradually increased [31]. 

Four active compounds m-Diethyl-benzene, p-Cymene, Carveol and Linalool were identified in GC-EAD assay. In previous studies, it has been shown that Linalool has a significant attraction to *A. gifuensis* [32,33], so the test of linalool was not carried out in this study. Moreover, MeSA could be used as an attractant to recruit *A. gifuensis* and other natural enemies [15,16,34]. However, female *A. gifuensis* did not show an obvious response to MeSA in the GC-EAD assay, which may be due to the decrease of antenna-activity with the increase in time. D-limonene, the precursor of the terpenes such as P-cymene, Carvol and Linalool synthesis, was increased after induction by MeSA. Therefore, Linalool, P-cymene and Carveol may all be synthesized with D-limonene as the precursor in wheat. Further research is needed to determine if D-limonene can effectively regulate the tri-trophic interactions among wheat plants, aphids and natural enemies.

In addition, the plant resistance to herbivores induced by chemical elicitors is affected by the genotype of the plant itself as well as the growth period and physiological condition of the plant when the elicitor is used [35]. Studies have confirmed that chemical elicitors can affect the chemical synthesis of plants and induce plants to produce more secondary metabolites, which may affect the allocation of carbon elements, inhibit plant growth and affect the yield and even quality of crops in the field [36,37]. Our experiments were conducted in laboratory with wheat seedlings, so the characteristics of the induced indirect defense under field conditions need to be investigated further.

In conclusion, this study suggests that the exogenous application of MeSA can induce an indirect defense response in wheat plants. Such general patterns of activity suggest the possibility of enhancing defense in crop plants via the development of plant defense elicitors. Given the mounting interest in manipulating plant semio-chemicals with the use of plant elicitors as a new tactic for protecting crops against insect pests, we suggest that MeSA could provide such a benign tool in wheat defense against aphids. Considered field application, the functional significance of VOCs induced by MeSA on predators of wheat aphids need to be further investigated. The effects and mechanisms of MeSA as a plant elicitor to enhance plant defenses against pests of other crops also need to be investigated.

## 4. Materials and Methods

### 4.1. Plant 

*Triticum aestivum* var. Jimai 22, were sown in plastic pots (10 cm in diameter, volume 500 mL) with organic matter nutritional soil (vermiculite, perlite and peat, mixed at a ratio of 1:1:4). The plastic pots with wheat seeds were kept in a climate chamber at 22 ± 1 °C, 60–70% r. h. and a L:D photoperiod of 14:10 h. The plants were grown without insecticide application. Plants with two developed leaves were used for behavioral assays and volatile chemical analyses [38].

### 4.2. Insects 

The initial colony of *A. gifuensis* was obtained from aphid mummies of *Myzus persicae* (Sulzer) provided by Yunnan Green Biological Technology Co., Ltd., Dali, Yunnan, China, and raised in a climate chamber on the same host and tobacco plants under the condition of 22 ± 1 °C, 60 ± 5% r. h. and a L:D photoperiod of 12:12 h for at least one generation. The new mummies were collected and placed into glass tubes (200 × 25 mm) covered by cotton wool and kept in the climate chamber at the same condition. The newly emerged adults were kept in new tubes per 24 h, and covered by cotton wool moistened with 10% honey solution and maintained for up to 48 h before the experiments [39]. All wasps used in this study were © and used only once. Before each trial, *A. gifuensis* adults were starved for 2 h to enhance their sensitivity to odors.

### 4.3. Chemicals 

MeSA (99.5%) and n-hexane (≥98%) were purchased from Kaitong Chemical Reagents Co., Ltd., Tianjin, China. p-Cymene, Carveol and m-Diethylbenzenel (≥98%) were purchased from Sigma Aldrich, Germany.

### 4.4. Plant Treatments 

Wheat seedlings were sprayed with an aqueous emulsion of MeSA. Spray treatments were carried out using a hydraulic nozzle mounted on a variable speed spray track at 1 m/s [40]. Plants were randomly allocated to one of the following treatments: (a) 0.5 mmol/L MeSA formulation (33.4 µL MeSA and 500 µL Ethylan BV in 500 mL deionized water); (b) 1 mmol/L MeSA formulation (66.8 µL MeSA and 500 µL Ethylan BV in 500 mL deionized water); (c) Control (500 μL Ethylan BV in 500 mL of deionized water). Ethylan BV was used to emulsify MeSA. All solutions should be used as soon as they were prepared. Wheat seedlings were sprayed twice, 24 h apart, until droplets flowed down. Then, they were covered with transparent plastic bags for 6 h to prevent MeSA volatilization. Wheat leaves were subjected to dynamic headspace volatile collection or olfactory assays after exposure to MeSA for 24, 48 and 72 h.

### 4.5. Volatile Organic Compound (VOC) Collection

Wheat plants used for VOCs collection were washed in advance. For each collection, 100 g wheat seedlings were enclosed in a 2000 mL conical bottle, with two collection ports at the top (one for inlet of air and the other for outlet). A small amount of deionized water was placed in the conical bottle to ensure the transpiration of the wheat seedlings. Air, purified by passing through an activated charcoal filter (BDH, 10–14 mesh, 500 g), was pushed into the vessel through the inlet port at 400 mL/min (flow rate controlled by a needle valve and measured by a flow meter), and then moved out of the jar into a Porapak Q (100 mg, 80/100 mesh, Supelco, Bellefonte, PA, USA). All connections were made with polytetrafluoroethylene (PTFE) tubing and sealed with PTFE tape. Porapak Q tubes were conditioned before use by washing with n-hexane (4 mL) and heated to 250 °C under a stream of purified nitrogen and kept for 2 h [40]. The collection of volatiles was carried out for 24 h with 3 replications. Trapped volatiles were eluted with 500 μL of n-hexane, then the solution was concentrated to 200 μL and stored at −80 °C until analysis [38].

### 4.6. Chemical Analysis 

An aliquot (1 μL) of headspace wheat seedlings volatile extract was analyzed by GC/MS on an Agilent 7890A coupled with Agilent 7000D (Agilent Technologies Co., Ltd, Santa Clara, CA, USA). The mass spectrometer was equipped with an Inert Cap 5MS/NP capillary column (5% diphenyl and 95% dimethylpolysiloxane, 30 m × 0.25 mm × 0.25 μm film thickness). Analysis was performed in the splitless mode using helium as carrier gas at a constant flow rate of 1 mL/min The oven temperature was maintained at 40 °C for 1 min, increased with a rate of 5 °C/min to 60 °C, held for 5 min. and then with a rate at 10 °C/min to 250 °C, held for 5 min, and spectra were recorded at 70 eV, source temperature 250 °C. Solvent delay was 5 min. Tentative identifications were made by comparison of spectra with mass spectral databases (NIST, Wiley). The relative content of volatiles was calculated by Agtqual software according to the relative peak area.

### 4.7. Gas Chromatography-Electroantennogram Detection (GC−EAD)

To determine which individual volatile compounds elicited female wasp antennal responses, the GC-EAD analysis was conducted using a gas chromatograph (GC). GC (7890B; Agilent, Santa Clara, CA, USA) was equipped with an HP-5ms Ultra Inert column (30 m × 250 mm ID and a film thickness of 0.25 mm, Agilent Technologies, Santa Clara, CA, USA) [41], respectively. For each run, a 4 μL sample was injected in splitless used as the mobile phase at a linear velocity of 40 cm/s, the oven initial temperature at 50 °C, ramped to 100 °C at 5 °C/min, held for 5 min, and then ramped-up to 250 °C at 10 °C/min. The compounds were carried to the antenna through a glass tube by a charcoal-filtered and humidified air stream at 0.5 m/s. The antenna was excised from a female *A. gifuensis* with fine forceps and the excised antenna was mounted on a microelectrode by using an electrode gel connected to a micromanipulator (MP-15; Syntech, Battaramulla, Sri Lanka). The stimulus controller unit (CS-55; Syntech, Battaramulla, Sri Lanka) provided a stable airflow by maintaining a flow rate of 20–30 mL/min. Signals were amplified with a USB acquisition controller (IDAC-2; Syntech, Battaramulla, Sri Lanka) and transferred to a computer. Data collection and processing were performed using GC-EAD 2010 software (Syntech, Battaramulla, Sri Lanka).

### 4.8. Comparative Electroantennogram (EAG) Responses to Selected Compounds 

To identify chemicals and different doses that may be potentially responsible for the attraction of *A. gifuensis*, the EAG responses of *A. gifuensis* to the candidate compounds (p-Cymene, Carveol, m-Diethyl-benzene) were tested. A wasp was frozen for around half a minute, and then one antenna was removed from the head and the tip of the antenna was removed. Each dissected antenna was immediately fastened with electrode gel onto two metal electrodes [42]. Each test requires 10 μL of the chemical to be applied to the filler paper (0.8 × 1 cm), then the filter is placed in a plastic Pasteur pipet (5 cm in length, inner diameter of 0.5 cm), the tip of the pipet inserted for about 2 mm into a small hole in the EAG device. The humidified air flow rate is 0.4 m/s by a stimulus controller, stimulation time 0.5 s. EAG responses to 10 μL n-hexane were tested as a control. EAG records of six antennae were required for each solution. Signals were stored and analyzed using EAG ver.2.5 software (Synthech, Hilversum, The Netherlands). 

### 4.9. Y-Tube Olfactometer Bioassay 

The olfactory responses of female *A. gifuensis* to wheat plants and the plant volatile collections were detected in a Y-tube olfactometer (diameter, 1.5 cm; common tube length, 20.0 cm; length of each arm, 15.0 cm, and both arms were extended at 75°). Wheat plants were put into two custom-made glass chambers (1.0 L capacity), each contained 20 wheat plants. For the responses to volatile collections, an aliquot (10 μL) of each test solution was applied to a filter paper strip (2 × 1 cm) and the solvent was allowed to evaporate (1 min) before inserting the strip into an odor-source glass bottle connected to one arm of the olfactometer. Three treatments (24 h after treated with 1 mmol/L MeSA, 0.5 mmol/L MeSA and control) were compared pairwise. Each group was tested with 30 female adults and individuals with no responses were discarded.

According to GC-EAD and EAG assays, three compounds p-Cymene, Carveol, and m-diethyl-benzene were selected to identify their functional significance for *A. gifuensis* attraction. Chemicals were prepared in different concentrations using n-hexane as control (0.01, 0.1, 1, and 10 μg/μL). An aliquot (10 μL) of each test solution was applied to a filter paper strip (2 × 1 cm) and the solvent was allowed to evaporate (1 min) before inserting the strip into an odor-source glass bottle connected to one arm of the olfactometer. The control glass bottle connected to the other arm of the olfactometer contained a filter paper strip treated with 10 μL of hexane. Each test of the chemical required 20 female adults and individuals with no responses were discarded. 

Airflow through the arms was 200 mL/min. To avoid interference from other light sources, the Y-tube olfactometer was held and placed inside a dark environment. Illumination was provided by a fluorescent bulb (ca. 100 lx). If an *A. gifuensis* adult crossed half the arm within 5 min, it was considered as having made a choice. Insects which did not make a choice within 5 min were considered as nonresponding [38]. After testing two insects, the two arms should be switched to avoid positional bias [42]. After testing five insects, the olfactometer was cleaned with anhydrous ethanol, rinsed with deionized water and dried in an oven.

### 4.10. Data Analysis

Data on EAG responses to synthetic chemical solutions were analyzed using analysis of variance (ANOVA followed by LSD test). Results of Y-tube olfactory bioassays were analyzed with a χ^2^ test to test the null hypothesis that there was no preference of adult *A. gifuensis* to odors of the selected chemicals. Individuals that did not make a choice were excluded from the statistical analysis.

From the VOC blends of wheat seedlings, 20 VOCs were provably identified. Given that each treatment has three independent biological replicates, a multivariate statistical technique called principal component analysis (PCA) was used to clarify how each of these 20 VOCs contributed to explaining the distinction in the blends produced by different wheat treatments.

## Figures and Tables

**Table 1:** Compounds identified from wheat plant volatile extracts via GC−MS.

No.	Library/ID (by Retention Time)	0.5 mmol/L	1 mmol/L	Control
1	β-pinene	+	+	
2	2,5-Dimethyl-1-hepten-4-ol		+	
3	3-Carene	+	+	+
4	Benzene,1-ethyl-2-methyl	+	+	+
5	6-methyl-5-Hepten-2-one	+	+	+
6	Mesitylene	+	+	+
7	m-Diethylbenzene	+	+	
8	D-Limonene	+	+	+
9	p-Cymene		+	
10	Carveol		+	
11	Nonanal	+	+	
12	Benzaldehyde,3-ethyl	+	+	
13	Levomenthol		+	
14	Dodecane,4,6-dimethyl	+	+	+
15	Linalool		+	
16	Methyl salicylate	+	+	
17	Decanal	+	+	
18	Tridecane	+	+	+
19	Tetradecane		+	
20	β-Acorenol		+	

Note: “+” means the presence of compounds in plant volatiles. 0.5 mmol/L and 1 mmol/L: Volatiles were identified at 24, 48, and 72 h after treatment with MeSA. Control: untreated wheat plant.

**Table 2:** The relative content analysis of volatiles.

Compounds	Control	0.5 mmol/L24 h	0.5 mmol/L48 h	0.5 mmol/L72 h	1 mmol/L24 h	1 mmol/L48 h	1 mmol/L72 h
3-carene	1.23 ± 0.55 b	1.77 ± 0.77 b	1.94 ± 0.41 b	0	3.65 ± 0.56 a	3.17 ± 0.60 a	2.93 ± 1.13 a
β-pinene	0	0	1.58 ± 0.28 a	1.99 ± 0.19 a	0	0	1.12 ± 0.03 a
6-methyl-5-Hepten-2-one	0	2.70 ± 0.30 b	1.52 ± 0.23 b	0	4.87 ± 1.36 a	4.22 ± 0.35 a	2.48 ± 0.49 b
D-limonene	12.96 ± 3.78 c	100 a	43.28 ± 1.84 b	100 a	70.38 ± 12.13 a	59.34 ± 7.89 ab	48.63 ± 10.12 b
m-Diethylbenzene	0	0	0	0	2.95 ± 0.95 a	1.32 ± 0.64 b	0
P-cymene	0	1.45 ± 0.01 a	1.49 ± 0.05 a	0	2.05 ± 0.34 a	1.67 ± 0.17 a	1.24 ± 0.04 a
Carveol	0	0	0	0	1.37 ± 0.037 a	1.38 ± 0.034 a	0
Nonanal	0	7.45 ± 0.92 a	4.59 ± 0.77 a	2.04 ± 0.35 b	4.31 ± 1.89 a	2.03 ± 0.55 b	4.45 ± 1.34 a
Linalool	0	0	0	0	3.39 ± 0.56 a	2.30 ± 0.32 a	0
MeSA	0	4.53 ± 0.92 c	3.18 ± 0.14 c	57.39 ± 8.83 b	68.10 ± 17.97 b	57.39 ± 8.83 b	100 a
Levomenthol	0	0	0	0	1.12 ± 0.03	0	0

Note: The compounds were ordered according to their increasing GC retention time. The time in the table refers to the time from the completion of treatment to the beginning of headspace collection. The relative content of volatiles was calculated by Agtqual software according to the relative peak area. Different letters indicate significant differences between treatments (*p* < 0.05) when the one-way ANOVA result was significant (*p* < 0.05, F-test).

## Data Availability

The data presented in this study are available on request from the corresponding authors.
